# Anti-tumor pharmacological evaluation of extracts from *stellera chamaejasme L* based on hollow fiber assay

**DOI:** 10.1186/1472-6882-14-116

**Published:** 2014-03-31

**Authors:** Xiaoni Liu, Qing Yang, Ganlin Zhang, Yujie Li, Ying Chen, Xiaogang Weng, Yajie Wang, Yiwei Wang, Xiaoxin Zhu

**Affiliations:** 1 Beijing Institute of Hepatology and Beijing YouAn Hospital, Capital Medical University, Beijing, 100069, China; 2 Institute of Chinese Materia Medica, China Academy of Chinese Medical Sciences, No 16 Nan Xiao Jie, Dong Zhi Men Nei, Dong Cheng Qu, Beijing, 100700, China

**Keywords:** *Stellera chamaejasme L*, Hollow fiber assay, Apoptosis, Caspase activity, Fas, TNF-α

## Abstract

**Background:**

*Stellera chamaejasme L*, a traditional Chinese herb, has long been used for treatment of various tumors in the Chinese population. In our previous study, we paid an attention to the cytotoxic and proapoptotic effects of *Stellera chamaejasme L* extracts (ESC, ESC-1 and ESC-2, the latter two were isolated from ESC) on 4 various tumor cells (NCI-H157, NCI-H460, BEL-7402 and SK-HEP-1) *in vitro*. ESCs showed significantly inhibitory effects on the 4 tumor cells. ESC-2 had the strongest inhibitory effect and the broadest sensitive cell spectrum. ESC-2 and ESC acted in a similar way against tumor cells, which suggested anti-tumor active fraction of ESC might exist in ESC-2. Here, we further observe the inhibitory and proapoptotic effects of *Stellera chamaejasme L* extracts *in vivo*.

**Methods:**

In this study, we used hollow fiber tumor model to evaluate the inhibitory and proapoptotic effects of *Stellera chamaejasme L* extracts. Apoptotic rates of the cancer cells retrieved from the hollow fibers were measured with flow cytometric analysis, caspase 3, 8, 9 enzyme activities were detected by colorimetric assay, Fas, Fas-L, TNF-R1 and TNF-α expression were determined with elisa assay and radioimmunoassay respectively.

**Results:**

The results showed that ESC, ESC-2 all had inhibitory effects on 4 tumor cells. According to the effect strength, dose and antitumor spectrum, the order of antitumor effects of ESCs was: ESC-2 > ESC > ESC-1. NCI-H460 cells were the most sensitive to ESCs. ESC, ESC-2 increased greatly the apoptotic rate and caspase 3, 8 enzyme activities in NCI-H460. ESCs had no significant effects on expression of Fas, Fas-L protein, but TNF-α/TNFR1 protein expression in NCI-H460 cells changed significantly after ESC and ESC-2 treatment.

**Conclusion:**

ESC-2 had the similar antitumor effect to that of ESC *in vivo* and further confirmed that ESC-2 may be the main antitumor active fraction of ESC, which was consistent with our previous results *in vitro*.

## Background

*In vitro* cytotoxicity experiment is the first step for screening or discovery of anti-tumor drug. It has the advantages of reducing substantially the amount of test drugs, determining the tumor specificity of the test drugs, simple experiment, low cost, etc. So, it is also easily adopted as the approach for pharmacological researchers to tracing active ingredients or fractions of most anti-tumor nature products, especially medicinal herbs. However, each herb in body is subjected to process of transport and metabolism, which makes the experimental results *in vitro* and *in vivo* always inconsistent. In particular, distinctive action characteristics of traditional Chinese herbs based on its integrity and interaction with the body determine the importance and necessity of investigating the efficacy *in vivo*. Therefore animal experiments must be conducted to verify the validity of the test samples, which is the critical step for active ingredients or fractions screening of traditional Chinese herbs. Xenograft is once the main the model for anti-tumor drug screening. But this kind of model is difficult to be as the large-scale screening method because it demands a large number of experimental animals and the test samples, consuming large amounts of time and manpower. Hollow fiber assay (HFA) is a high-throughput *in vivo* screening system built up by US National Cancer Institute (NCI) in 1995 [1] and has become one of the conventional anticancer drug screening method in NCI, which make it become reality to screen antitumor agents in large scale [2]. Because HFA can provide the real *in vivo* environment,in accordance with the action characteristics of natural products involving traditional Chinese herbs, HFA has potential broad application prospects in antitumor research field of traditional Chinese herbs and other natural products [3]. Moreover, a large number of research results showed that HFA was also an effective method to quickly research pharmacodynamics (e.g., Pervilleines B and C [4], Paclitaxel and Pacliex [5], C1311 and SJG-13 [6], ASI-145 [7], YSL [8], TAS-102 [9]) and mechanism (e.g., PCNA and Rb [10], p16 [11], NF-kappa B [12]) of antitumor drugs.

In our previous study, we paid attention to the cytotoxic and proapoptotic effects of *Stellera chamaejasme L*(a traditional Chinese herb) extracts (ESC, ESC-1 and ESC-2 were obtained with special techniques described as Materials and Methods below) on 4 various tumor cells (two lung cancer cell lines: NCI-H157 and NCI-H460; two liver cancer cell lines: BEL-7402 and SK-HEP-1) *in vitro*. The results showed ESC, ESC-1, ESC-2 all had inhibitory effects on the 4 tumor cells. On 72 hours, inhibition rates of 100, 200 μg/mL ESCs on the 4 tumor cells were 64.82%-92.27%. ESC-2 had the strongest inhibitory effect and the broadest sensitive cell spectrum. GI_50_, TGI and LC_50_ of ESC-2 were similar to those of ESC. These results suggested that antitumor effects of ESC-2 and ESC acted in a similar way, anti-tumor active fractions of ESC may exist in ESC-2 [13]. ESC and ESC-2 could induce the apoptosis by activating the key proteins of tumor apoptosis pathway in NCI-H460 cells [14,15]. Here, we further observe the inhibitory and proapoptotic effects of *Stellera chamaejasme L* extracts using HFA to confirm the *in vitro* effects of ESCs.

## Methods

### Preparation of *Stellera chamaejasme L* extracts

Process of ESC (extract of *Stellera chamaejasme L*) was given in another paper [14], ESC was mixed with reversed silica, filtered through a C18 column and eluted with 70%, 100% methanol respectively and the final samples were ESC-1 and ESC-2. Part components of ESCs were determined by 2695-996 Waters Alliance HPLC and Finnigan TSQ mass spectrometer to control the quality of ESCs (By Xiao HB, Dalian Institute of Chemical Physics, Chinese Academy of Sciences). The primary plant of *Stellera chamaejasme L* was identified by He XR at Institute of Chinese Materia Medica, China Academy of Chinese Medical Sciences where all testing samples were deposited.

### Materials and reagents

RPMI-1640 medium, fetal bovine serum (FBS), penicillin-streptomycin and trypsin- ethylenediaminetetraacetic acid(EDTA)were purchased from Gibco (Grand Island, NY, USA). 3-(4,5-Dimethylthiazol-2-yl)-2,5-diphenyltetrazolium bromide (MTT), dimethyl sulfoxide (DMSO) and protamine were provided by Sigma Chemical Co. (St. Louis, MO, USA). 5-fluorouracil (5-FU) injection was from Shang Hai Xu Dong Hai Pu Pharmaceutical Co. Ltd, Shanghai, China. Acutase was purchased from EBioScience (San Diego, CA, USA). Caspase-3,-8 colorimetric protease assay kits were purchased from Chemicon International, Inc. (Temecula, CA, USA). Fas, Fas-L, tumor necrosis factor receptor-1 (TNF-R1) ELISA kits were from R&D Systems (Minneapolis, MN, USA). Tumor necrosis factor-α (TNF-α) radioimmunoassay kit was from the radioimmunoassay institute of General Hospital of PLA, China. The biocompatible modified polyvinylidene difluoride (mPVDF) hollow fibers (CellMaxs Implant Membranes) with a Mr 500 kDa cutoff and 1.0 mm inner diameter were purchased from Spectrum Laboratories Inc. (Breda, The Netherlands). All the other chemicals used, unless otherwise stated, were obtained from Sigma Chemicals.

### Animals and animal ethical statement

Six to eight weeks old male Nu/Nu Nude Mice (from Beijing Vital River Laboratory Animal Technology Co. Ltd) were raised and maintained under specific pathogen free (SPF) sterile condition. All studies involving animals were conducted according to the welfare and ethical guidelines for experimental animal drafted by Beijing Experimental Animal Management Office (Executed on January 1, 2006) and approved by Committee for Control and Supervision of Experiments on Animals of Institute of Chinese Materia Medica, China Academy of Chinese Medical Sciences.

### Cell culture

Two non-small-cell lung carcinoma cell lines of NCI-H157, NCI-H460 were purchased from the Cell Culture Center of the Chinese Academy of Medical Science. Two liver cancer cell lines of BEL-7402, SK-HEP-1 were obtained from Cell Bank, Shanghai Institutes for Biological Sciences, Chinese Academy of Sciences. Cells were grown in 1640 medium containing 10% (v/v) FBS and incubated at 37°C in a 5% CO_2_ humidified atmosphere.

### Preparation and *in vivo* implantation of the hollow fibers

The hollow fiber tumor models were made by referring to the method of Hollingshead MG [1]. Hollow fibers were flushed with 70% ethanol solution followed by distilled water for hydration, then autoclaved (121°C for 40 min) to sterilize. The sterilized fibers were stored in the tray at 4°C to wait for to be used. Cells were cultured in accordance with the method described above. Cell density was controlled at 70-90% confluence at the day of the implantation. Cells were digested conventionally, adjusting the cell concentration in the range of 1 × 10^6^-10 × 10^6^/ml. Cell counts and activity identification were used trypan blue exclusion method. Adding an appropriate amount of 1640 medium containing 20% FBS to adjust the cells to a desired concentration, hollow fibers were removed from the tray and placed on the work surface flushed with cold fresh medium. Cells were injected into the fibers with a syringe (18 gauge needle) and fibers were sealed every 2 cm with heat-sealer for 3-5 seconds. Fibers were cut off in the middle of the heat seal segments. Fibers were put into 6-well plates preloaded with medium to culture overnight. Every nude mouse was anesthetized with sodium pentobarbital and the abdomen was incised, inserting the hollow fibers into abdominal cavity. After abdominal incision was sutured, a small incision was made in the back neck and hollow fibers were placed subcutaneously through the transplant casing.

### Cytotoxic assessment of ESCs

45 male nude mice (body weight range was 18-20 g) were made into hollow fiber tumor models according to the method above. Every mouse was loaded with 4 tumor cells: NCI-H157, NCI-H460, BEL-7402 and SK-HEP-1. These mice were divided into nine groups according to the weight: control (saline), solvent (10%DMSO), 20 mg/kg 5-Fu, 4.5 mg/kg ESC, 3.0 mg/kg ESC, 25 mg/kg ESC-1, 16.75 mg/kg ESC-1, 1.5 mg/kg ESC-2, 1.0 mg/kg ESC-2. The mice were treated on 4 day by intraperitoneal administration with ESCs (The doses of ESCs were based on the results of their maximum tolerated dose (MTD) experiments, the high dose = [MTD × 1.5]/4 and the low dose = 0.67 × the high dose [2]). The mice were sacrificed at day 8 and the fibers were retrieved from the mice. Excess host tissue was removed and the fibers were transferred into prewarmed medium and incubated for at least 1 h to be waited for pharmacodynamic analysis. The MTT assay was used to determine cytotoxicity of the cancer cells to the ESCs after isolation of the fibers. The fibers were placed into 2 ml of fresh, prewarmed 1640 medium in six-well plates and allowed to equilibrate for 30 min at 37°C. Prewarmed 1640 medium (1.5 ml) containing 1 mg MTT/ml was added to each well. After a 4 h incubation at 37°C in a 5% CO_2_ humidified atmosphere, the culture medium was aspirated and 2 ml normal saline containing 2.5% protamine sulphate was added into each well. The fibers were stored at 4°C for 24 h. Each fiber was transferred to 24-well plates after being removed debris outside of the fibers. The fibers were cut into half and dried at room temperature (RT). 250 μl DMSO was added into each well to dissolve formazan crystals for 4 h at RT on a rotating platform while protected from the light. Aliquots of 150 μl of extracted solution were transferred to individual wells in 96-well plates and absorbance value was measured at 490 nm using a spectrophotometer (ELX800 type, BIO-TEX Instruments, INC, Winooski, VT, USA). The inhibition rate (IR) was calculated as follows.

$$\mathrm{Inhibition}\;\mathrm{rate}\left(\%\right)=1-\frac{OD_{\mathrm{treatment}}-OD_{\mathrm{pre}‐\mathrm{implantation}}}{OD_{\mathrm{solvent}}-OD_{\mathrm{pre}‐\mathrm{implantation}}}$$

### Determination of apoptotic effects induced by ESCs

36 male nude mice (body weight range was 18-20 g), according to the above method, were charged hollow fibers with NCI-H460 lung cancer cells intraperitoneally and subcutaneously. Mice were divided into control, 20 mg/kg 5-Fu, 4.5 mg/kg ESC, 3.0 mg/kg ESC, 1.5 mg/kg ESC-2, 1.0 mg/kg ESC-2. The administration method was same as that mentioned above. On the collection day, the fibers were removed from sacrificed mice and transferred to 6-well plates added with 2 ml preheating acutase to digest for 30 min. Then the cells were flush out from fibers to be wait for detection of indexes related to apoptosis.

### Measurement of apoptotic rate

The cancer cells were retrieved from the fibers and prepared for flow cytometric analysis to measure apoptotic rate. Briefly, the cells were washed with phosphate balanced solution (PBS) and fixed with 70% ethanol for 30 min on ice. After centrifugation, the pellet was resuspended in 400 ml hypotonic propidium iodide (PI) solution (containing 0.5 mg/ml Ribonuclease, 50 mg/ml PI, 1 mg/ml sodium citrate, 1 ml/ml Triton X-100 in saline) and incubated on ice in the dark for at least 15 min. Then the cells were analysized by flow cytometer (Becton-Dickinson, USA).

### Detection of caspase activities

Caspase 3, 8, 9 activities were measured with caspase assay kits. 1 × 10^6^ cells were collected from fibers and lysised with lysate on ice for 10 min and centrifuged at 20,000 g for 15 min. The supernatant was transferred to a pre-cooled centrifuge tube and determined the activities of caspases immediately. Reaction system was set according to kits and incubated at 37°C for 120 min. Absorbance value was measured at 405 nm when the reaction system color obviously changed. If the color did not change significantly, incubation time of reaction system may be extended, even overnight incubation. Deference of A405 absorbance between treatment and the control was p-nitroaniline (pNA) absorbance of sample catalyzed by caspases. Catalytic amount of pNA of can be calculated through the pNA standard curve. Enzyme activity unit was defined as the amount of caspase enzyme catalyzing 1 nmol pNA substrate when the substrate was saturation. Each sample protein concentration was determined by Bradford method to calculate the enzyme activity in unit weight of protein of sample.

### Expression of Fas, Fas-L, TNF-R1 with Elisa assay

Cells removed from fibers were collected after conventional digestion and cleavage. The standard was diluted according to the Elisa kit. 100 μl standard or each test sample was added into wells of reaction plates and incubated for 120 min at 37°C. Plates were washed with washing liquid 5 times, and then first antibody working solution of 100 μl was added and incubated for 60 min at 37°C. Plates were washed another time. 100 μl Horseradish Peroxidase (HRP) working solution was added and incubated for 30 min. Plates were washed again. 100 μl substrate working solution was added and reacted in the dark for 15 min at 37°C, and then 100 μl of Stop Solution was added. Absorbances of samples were measured at 450 nm in microplate reader within 30 min.

### Expression of TNF-α with radioimmunoassay

Cells flushed from the hollow fibers were washed with PBS, and then cells were lysised with ultrasonic cell crasher. Using liquid competitive inhibition principle, the cell samples were determined with balance method. The samples or standard and limited antisera or antigen together generated competitive binding reaction. After the reaction, added immune separating agent to isolating the antigen-antibody complex, radioactivity of compound was determined and the standard combination rate was calculated. Make the standard curve and determine the sample concentration.

### Statistical analysis

All results were expressed as mean ± SD. One way analysis of variance (ANOVA) with multiple comparison tests was used. P < 0.05 was considered as significant. Statistical tests were performed using SPSS version 12.0.

## Results

### Comparisons of anti-tumor activities of ESCs based on HFA

**Figure 1:**
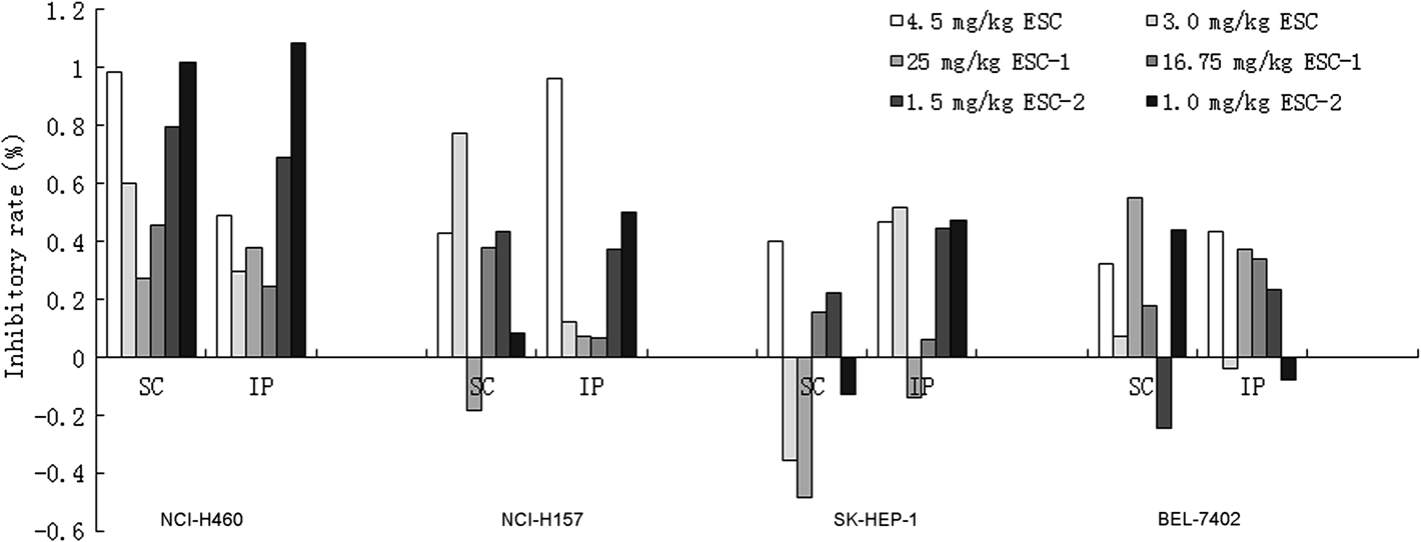
**Antitumor activities of ESCs on 4 tumor cell lines (NCI-H460, NCI-H157, SK-HEP-1 and BEL-7402) based on hollow fiber assay.** SC: subcutaneous fibers, IP: intraperitoneal fibers.

For the NCI-157 cells: In the SC (subcutaneous) fibers, the IRs of 4.5,3.0 mg/kg ESC and 1.5 mg/kg ESC-2 were 43.05%, 77.15% and 43.13% respectively; In the IP (intraperitoneal) fibers, the IRs of 4.5 mg/kg ESC and 1.0 mg/kg ESC-2 were 96.33% and 49.78% respectively. For the NCI-H460 cells: In the SC fibers, the IRs of 4.5,3.0 mg/kg ESC, 1.5,1.0 mg/kg ESC-2 and 16.75 mg/kg ESC-1 were 98.62%, 60.01%, 79.71%, 100.84% and 45.54% respectively; In the IP fibers, the IRs of 4.5 mg/kg ESC and 1.5,1.0 mg/kg ESC-2 were 48.70% and 68.71%,108.34% respectively. For the Bel-7402 cells, In the SC fibers, the IRs of 1.0 mg/kg ESC-2 and 25 mg/kg ESC-1 were 43.91% and 55.03% respectively; In the IP fibers, the IR of 4.5 mg/kg ESC was 43.38%. For the SK-HEP-1 cells: In the SC fibers, the IR of 4.5 mg/kg ESC was 40.24%; In the IP fibers, the IRs of 4.5, 3.0 mg/kg ESC, 1.5,1.0 mg/kg ESC-2 were 46.78%, 52% and 44.42%, 47.33%. The results showed that ESC and ESC-2 have obvious toxicity to 4 tumor cells in hollow fibers loaded in nude mice (see Figure 1).

### Apoptosis induced by ESCs in NCI-H460 cells from the implanted hollow fibers

**Figure 2:**
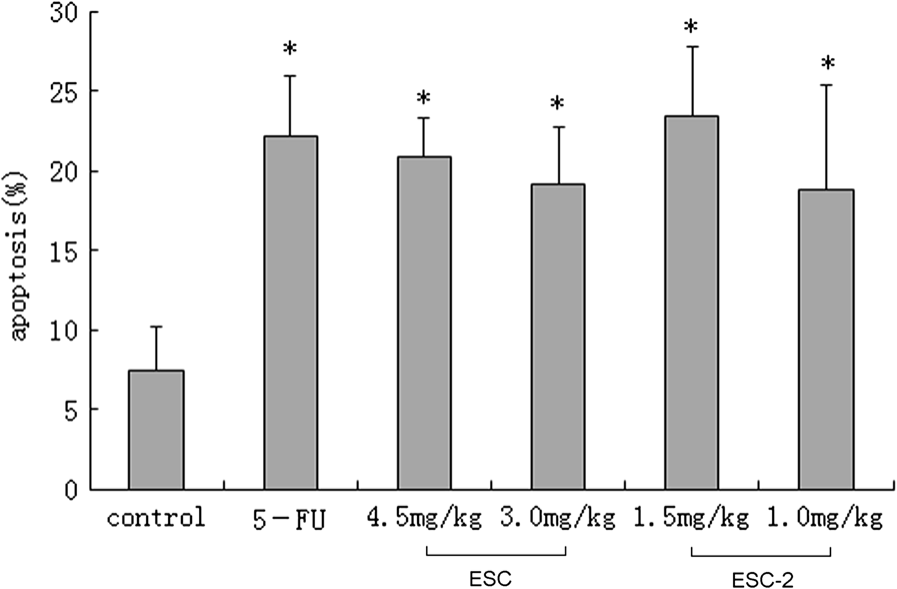
**Apoptosis induced by ESCs in NCI-H460 cells from the implanted hollow fibers in vivo.** The data were expressed as mean ± SD (n = 12 per group). *p < 0.05, significant *versus* control group.

Retrieval of the cells from the implanted fibers enabled to evaluate apoptosis induction. The cells retrieved from the fibers in control animals contained on average 7.40 ± 2.78 apoptotic cells. Otherwise, the cells retrieved from the fibers in 4.5, 3.0 mg/kg ESC and 1.5,1.0 mg/kg ESC-2 treatment groups contained on average 20.86 ± 2.39, 19.09 ± 3.60, 23.35 ± 4.45, 18.76 ± 6.58 apoptotic cells respectively, about 2.54 to 3.16 fold increase to that of control group (see Figure 2).

### Influence of ESCs on caspase 3, 8, 9 activities in NCI-H460 cells from the implanted hollow fibers

**Figure 3:**
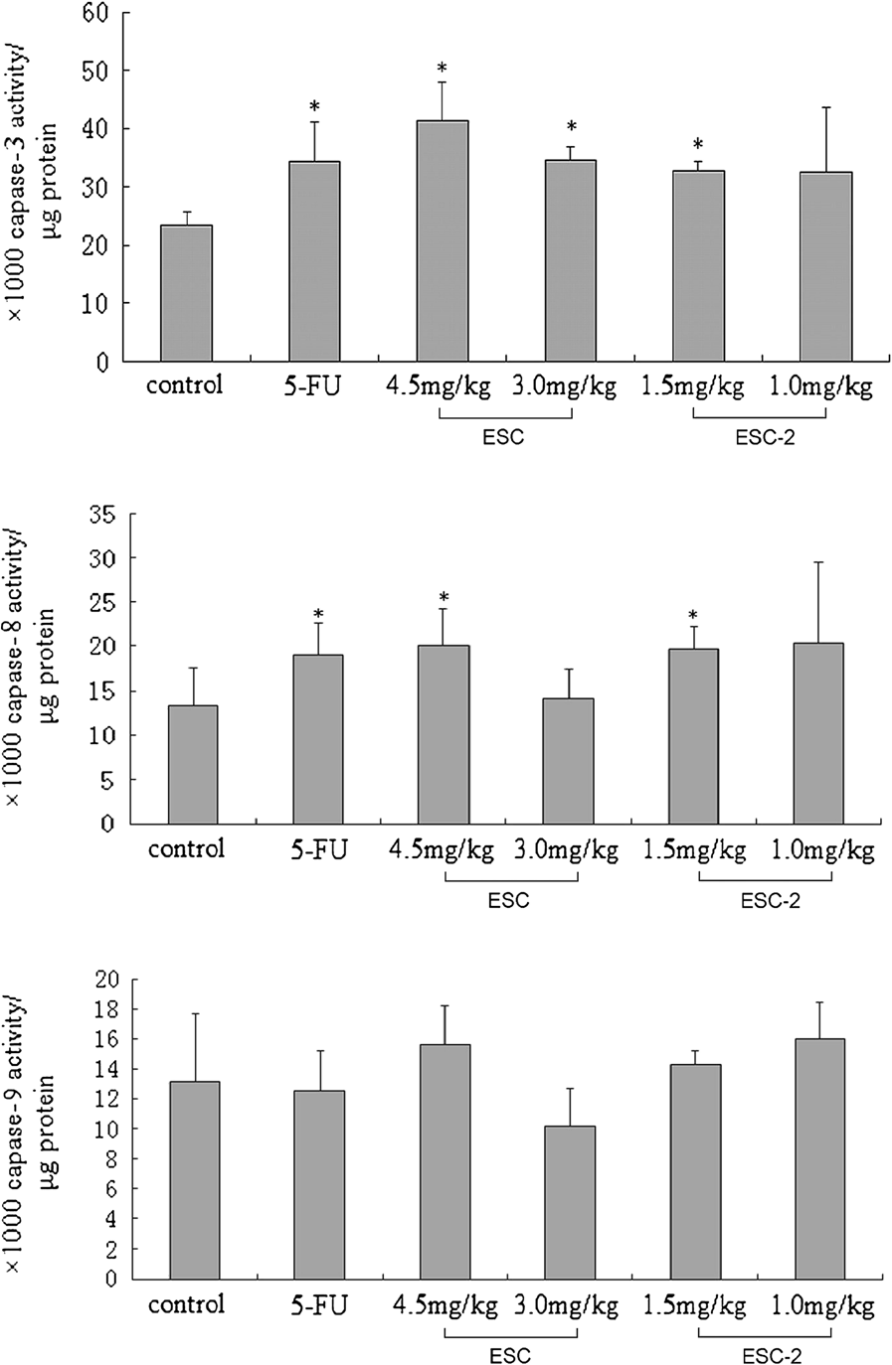
**Influence of ESCs on activities of caspase 3, 8, 9 in NCI-H460 cells from implanted hollow fibers.** The data were expressed as mean ± SD (n = 6 per group). *p < 0.05, significant *versus* control group.

4.5, 3.0 mg/kg ESC and 1.5 mg/kgESC-2 can significantly improve caspase 3 enzyme activity of NCI-H460 cells and increase 1.76,1.47 and 1.41 folds (41.17 ± 6.74, 34.49 ± 2.40,32.80 ± 1.59) respectively compared to that of control group (23.32 ± 2.26); 4.5 mg/kg ESC and 1.5 mg/kg ESC-2 can also significantly improve caspase 8 enzyme activity of NCI-H460 cells and increase 1.49 and 1.47 folds (19.98 ± 4.19,19.69 ± 2.54,) compared to that of control group (13.40 ± 4.17); but ESCs did not increase the caspase 9 enzyme activity of NCI-H460 cells (see Figure 3).

### Influence of ESCs on expression of Fas and Fas-L in NCI-H460 cells from the implanted hollow fibers

**Figure 4:**
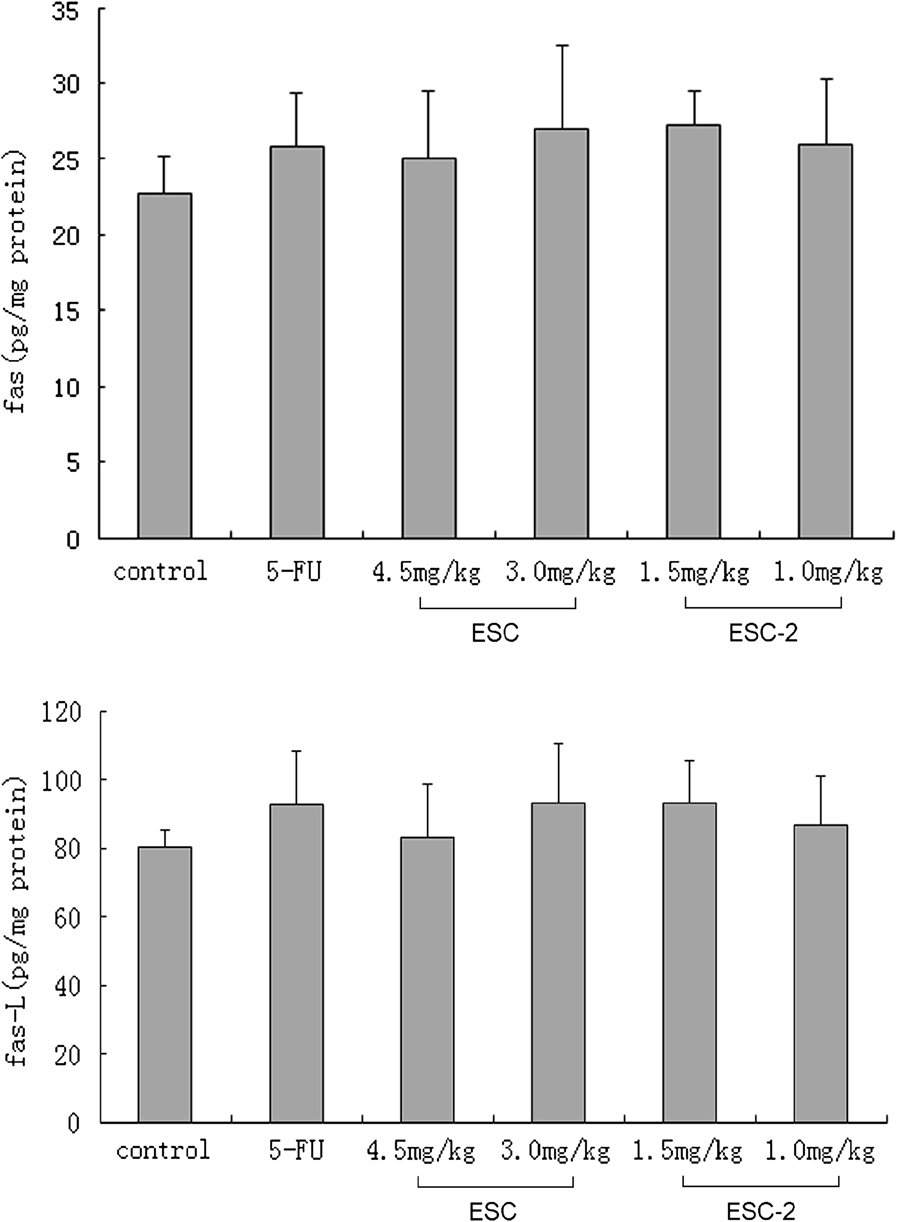
**Influence of ESCs on expression of Fas and Fas-L in NCI-H460 cells from implanted hollow fibers.** The data were expressed as mean ± SD (n = 6 per group). ESCs treatment groups has no significant change *versus* control group.

Compared with the control group, Fas and Fas-L expression of NCI-H460 cells in all ESCs groups has no significant increase (see Figure 4).

### Influence of ESCs on expression of TNF-α and TNFR1 in NCI-H460 cells from the implanted hollow fibers

**Figure 5:**
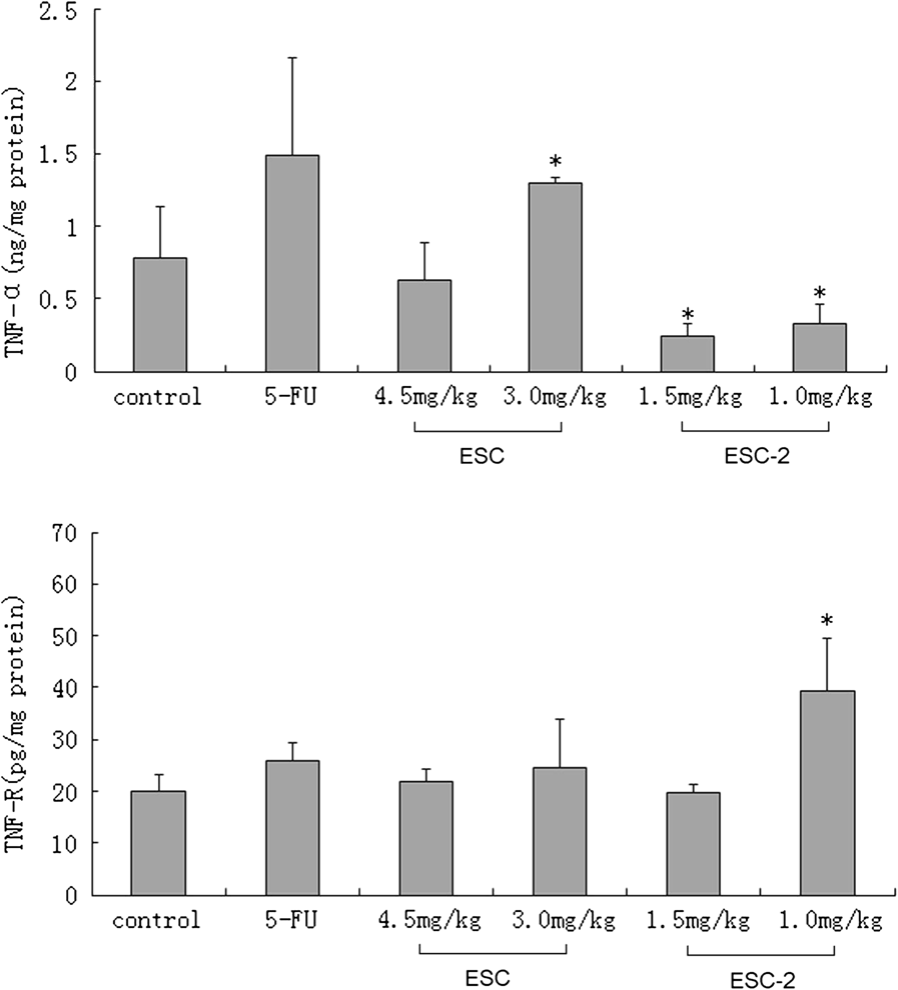
**Influence of ESCs on expression of TNF-α and TNFR1 in NCI-H460 cells from implanted hollow fibers.** The data were expressed as mean ± SD (n = 6 per group). *p < 0.05, significant versus control group.

3.0 mg/kg ESC(1.29 ± 0.03) significantly improved expression of TNF-α in NCI-H460 cells, whereas 1.5, 1.0 mg/kg ESC-2 (0.24 ± 0.09,0.33 ± 0.13) decreased significantly in NCI-H460 cell compared with the control group (0.78 ± 0.36); 1.0 mg/kg ESC-2 (39.10 ± 10.15) increased significantly TNFR1 expression in NCI-H460 cell compared with the control group(20.07 ± 3.02) (see Figure 5).

## Discussion and conclusions

In this study, we used hollow fiber assay (bearing NCI-H157, NCI-H460, BEL-7402, SK-HEP-1 4 tumor cell lines, subcutaneously and intraperitoneally implanted sites for each cell line for every animal)to evaluate the anti-tumor pharmacological effects of *Stellera chamaejasme L* Extracts *in vivo* and traced the main fragments of *Stellera chamaejasme L.* Of all the ESCs treatment mice, the average body weight losses were all <20% with no drug-related deaths, suggesting that all the experiment mice could tolerate ESCs during the experimental process.

It was considered positive that *in vivo* inhibition rate was more than 40% according to evaluation standard of *in vivo* antitumor nature product crude extract [16]. The hollow fiber assay results showed that ESC, ESC-2 had obvious *in vivo* cytotoxic effect and both produced >40% growth inhibition on four tumor cell lines, ESC-1 only one dose had certain inhibition on NCI-H460 and BEL-7402 cells. Especially, the cell killing effect (a reduction in cell mass below the input mass and inhibition rate was more than 100%) was observed in NCI-H460 cell line in mice of ESC-2 group. Therefore, the order of antitumor effects of ESCs was comprehensively evaluated according to the effect strength (ESC-2 had cell killing effect), effect dose (administration dose of ESC-2 was 1/3 of ESC) and the antitumor spectrum (ESC-2 had obvious inhibitory effects on 4 cell tumor lines): ESC-2 > ESC > ESC-1.

Comparison of sensitivity of ESCs to tumor cells, NCI-H460 cell line was the most because that all ESCs had inhibition on it. ESC-2 showed the strongest inhibition and inhibition rates of two sites (SC and IP) of NCI-H460 cells were more than 100%. Follow the ESC-2 was ESC and inhibition rate of two sites (SC and IP) were nearly 100%. Furthermore, ESC-1 showed certain inhibition in SC site on NCI-H460 cells.

In our previous *in vitro* experiment, we found that ESCs could induce the tumor cells apoptosis by activating death receptor pathway. Therefore we selected the most sensitive NCI-H460 cell line to further confirm the apoptotic effect of ESCs *in vivo* based on HFA. The flow cytometry results showed that 4.5 mg/kg ESC, and 1.5 mg/kg ESC-2 increased significantly apoptosis rates in NCI-H460 cells *in vivo*. Enzymatic activity assay also confirmed the ESC and ESC-2 could significantly increase the activities of caspase 3 and caspase 8 in NCI-H460 cells. These results suggested that ESC and ESC-2 induce tumor cell apoptosis through activation signal of the death receptor pathway *in vivo* which was consistent with the *in vitro* results [14,15].

The death receptor families contained Fas, TNFR1, death receptor (DR3, DR5), TNF related apoptosis inducing ligand receptor (TRAIL-R1, TRAIL-R2), etc. [17]. Which pathway would the ESCs acted on?

We tested the classic death receptors, Fas, TNFR1 and their ligands Fas-L, TNF-α. The Fas/Fas-L system was a key regulator of apoptosis. The results showed that ESC, ESC-2 had no significant effects on Fas/Fas-L pathway which was not consistent with the *in vitro* results. Maybe after the metabolism *in vivo*, the targets of ESCs changed. TNFR1/ TNF-α signal system was another important death receptor pathway which was not observed in our *in vitro* experiment. TNF-α was a multifunctional cytokine, mainly involving in inflammation and immune responses. It had a direct cytotoxic effect on tumor cells and could cause tumor necrosis. Recent researches had demonstrated that TNF-α inhibited certain tumor cell proliferation and induced apoptosis of tumor cells [18]. But few study focused on the role of endogenous tumor TNF-α, some scholars believed that the endogenous TNF-α could promote tumor cells to produce oxygen free radical ions, causing DNA damage or fracture [19,20] and was conducive to the treatment of tumors. Down-regulation of endogenous TNF-α to increase sensitivity of exogenous TNF-α (produced by other cells) on tumor cells was believed to be another reason of TNF-α killing tumor cells [21]. Other reports showed that TNF-α produced by tumor cells would injury normal tissue, promote tumor metastasis and related to many paraneoplastic syndrome (such as hypercalcemia, lipemia and tumor associated cachexia) [22,23]. Visibly, endogenous TNF-α is a double-edged sword. In this experiment, we observed that regulation roles of ESC, ESC-2 on endogenous TNF-α expression of tumor cells were opposite: 3.0 mg/kg ESC significantly upregulated whereas 1.5, 1.0 mg/kg ESC-2 downregulated the expression of TNF-α. ESC might upregulate the level of TNF-α to direct its effect of killing tumor and ESC-2 may downregulate endogenous TNF-α to enhance sensitivity of exogenous TNF-α to tumor cells. The possible and reasonable explanation for the results was that because ESC-2 was isolated from ESC, the target ingredients to TNF-α of ESC-2 may be changed.

TNFR1, another death receptor protein, combining with TNF-α might induce typtical apoptosis of caspase dependent pathway. On the other hand, this combination might activate the nuclear factor kappa-light-chain-enhancer of activated B cells (NF-κB) and c-Jun-N terminal kinase (JNK) signal pathway by recruitment of TNF receptor-associated factor (TRAF2) and Receptor interacting protein (RIP) [24-26]. 1.0 mg/kg ESC-2 obviously increased TNFR1 expression which suggested that ESC-2 inducing apoptosis of NCI-H460 cells might be related to upregulation of TNFR1 receptor.

The results above showed ESC-2 had the similar antitumor effect to that of ESC *in vivo* and further confirmed that ESC-2 may be the main antitumor active fraction of ESC, which was consistent with the *in vitro* result.

## Competing interests

The authors declare that they have no competing interests.

## Authors’ contributions

XL was the main experimental investigator and had drafted the manuscript. QY, GZ and YL helped to establish the hollow fiber tumor model. YC and XW helped to complete the experiments of caspases acticities and elisa assay. YW helped to correct the language of the manuscript. YW helped to analysis the data. XZ supervised the study and the manuscript. All authors read and approved the final manuscript.

## Pre-publication history

The pre-publication history for this paper can be accessed here:

http://www.biomedcentral.com/1472-6882/14/116/prepub
